# Supplementary material for: Comparative Analysis of Biofilm Formation and Antibiotic Resistance in Five ESKAPE Pathogen Species from a Tertiary Hospital in Bangladesh
Source: Antibiotics (Basel). 2025 Aug 20;14(8):842. doi: 10.3390/antibiotics14080842 (PMC12382965; doi:10.3390/antibiotics14080842)
Supplement: Supplementary file 1 [file antibiotics-14-00842-s001.zip › antibiotics-3617940-supplementary.pdf]

Supplementary Material

Supplementary Table S1 Primer list and PCR condition

Gene	Primers (5′ - 3′)	Tm (°C)	Product size (bp)	PCR condition	Ref.
nuc	F: GCGATTGATGGTGATACGGTT<br>R: AGCCAAGCCTTGACGAACTAAAGC	55	270	94°C (5 min) +35X [94°C (1 min) +55°C (30s) +72°C (1 min)] +72°C (7 min)	34
blaOXA-51	F: TAA TGC TTT GAT CGG CCT TG<br>R: TGG ATT GCA CTT CAT CTT GG	57	501	94°C for 3 min, and then 35 cycles at 94°C for 45 s, at 57°C for 45 s, and at 72°C for 1 min, followed by a final extension at 72°C for 5 min.	35
ddl E. feacium	F: GCAAGGCTTCTTAGAGA<br>R: CATCGTGTAAGCTAACTTC	50	550	94°C for 5 min, 30 cycles of 94°C for 30 sec, T m for 90 sec, and 72°C for 60 sec with a final extension of 72°C for 10 min.	36
ddl E. feacalis	F: ATCAAGTACAGTTAGTCTT<br>R: ACGATTCAAAGCTAACTG	50	941	Same as *ddl E. feacium*	36
oprL	F: ATGGAAATGCTGAAATTCGGC<br>R: CTTCTTCAGCTCGACGCGACG	57	504	30 cycles: 40s 94°C, 40 s 57°C, 50 s 72°C	37
IA-ITS	F: ATT TGA AGA GGT TGC AAA CGA T<br>R: TTC ACT CTG AAG TTT TCT TGT GTT C	57	130	94 °C (10 min) + 35X [94 °C (30 s), 57 °C (20 s) +72 °C (20s)] +72 °C (10 min)	38

mecA	F: AAAATCGATGGTAAAGGTTGGC<br><br>R: AGTTCTGCAGTACCGGATTTGC	50	533	(95°C, 4min) + [(94°C, 60s) + (50°C, 30s) + (72°C, 1.5min)] ×30 + (72°C, 10 min)	42
mecC	F: GAAAAAAAGGCTTAGAACGCCTC<br><br>R: GAAGATCTTTTCCGTTTTCAGC	59	138	(94°C, 5min) + [(94°C, 30s) + (59°C, 60s) + (72°C, 60s)] ×30 + (72°C, 10 min)	42
vanA	F: CATGAATAGAATAAAAGTTGCAATA<br><br>R: CCCCTTTAACGCTAATACGATCAA	54	1030	95 °C for 15 min, followed by 35 cycles of 1 min at 94 °C, 30 sec at 54 °C, and 1 min at 72 °C. A final extension step was performed at 72 °C for 10 min.	43
vanB	F: ATGGGAAGCCGATAGTC<br><br>R: GATTTCGTTCTTCGACC	54	435	Same as *vanA*	43
clfA	F: CGCCGGTAACTGGTGAAGCT<br><br>R: TGCTCTCATTCTAGGCGCACTT	55	314	(95°C, 5min) + [(95°C, 30 s) + (55°C, 30 s) + (72°C, 45 s)] ×30+(72°C, 10 min)	48
clfB	F: ATGATCTTGCTTGCGTT<br><br>R: CCGATTCAAGAGTTACACC	47	215	(95°C, 5min) + [(95°C, 30 s) + (47°C, 30 s) + (72°C, 45 s)] ×30+(72°C, 10 min)	48
fnbA	F: GCGGAGATCAAAGACAA<br><br>R: CCATCTATAGCTGTGTGG	48	1279	(95°C, 5min) + [(95°C, 30 s) + (48°C, 30 s) + (72°C, 45 s)] ×35+(72°C, 10 min)	48

cna	F: AATAGAGGCGCCACGACCGTATAC<br><br>R: GTGCCTTCCCAAACCTTTTGAGCA	54	155	(94°C, 5min) + [(95°C, 30 s) + (54°C, 30 s) + (72°C, 3min)] ×35+(72°C, 10 min)	48

bbp	F: AAC TAC ATC TAG TAC TCA ACA ACA G<br>R: ATG TGC TTG AAT AAC ACC ATC ATC T	53	575	95°C for 5 min, 35 cycles of 95°C for 30 sec, T m for 30 sec, and 72°C for 45 sec with a final extension of 72°C for 10 min.	49
IsdA	F: CTG CGT CAG CTA ATG TAG GA<br>R: TGG CTC TTC AGA GAA GTC AC	52	332	Same as *bbp*	50
IsdB	F: ACG AGA GTT TGG TGC GCT AT<br>R: GTT GAG GCC CCT ACT TCT GA	55	192	35 cycles of 95°C for 30°C sec, 55°Cfor 1 min, and 72°C for 2 min in with a final extension of 72°C for 10 min.	51
SdrD	F: CGG AGC TGG TCA AGA AGT AT<br>R: TGC CAT CTG CGT CTG TTG TA	52.3	500	Same as *IsdA*	50
SdrE	F: AGA AAG TAT ACT GTA GGA ACT G<br>R: GAT GGT TTT GTA GTT ACA TCG T	50	433	Same as *IsdA*	52
hyl	F: ACAGAAGAGCTGCAGGAAATG<br>R: GACTGACGTCCAAGTTTCCAA	56	276	An initial activation step at 95°C for 15 min, during which the HotStarTaq DNA polymerase is activated, was followed by 30 cycles of denaturation (94°C for 1 min), annealing (56°C for 1 min), and extension (72°C for 1 min), followed by one cycle consisting of 10 min at 72°C.	53
esp	F: AGATTTCATCTTTGATTCTTGG<br>R: AATTGATTCTTTAGCATCTGG	56	510	Same as *hyl*	53
asa1	F: GCACGCTATTACGAACTATGA<br>R: TAAGAAAGAACATCACCACGA	56	335	Same as *hyl*	53
gelE	F: TATGACAATGCTTTTTGGGAT<br>R: AGATGCACCCGAAATAATATA	56	213	Same as *hyl*	53
acm	F: GGCTAGTCGTTACAAATGAG<br>R: ATTTTATTCTTTTGATTTCAGTC	58	655	initial denaturation (94° C for 5 min), followed by 25–30 cycles of denaturation (94° C for	54

				35–45 s), annealing (50–68° C, from 45 s to 1 min) and extension (72° C, from 45 s to 1 min 35 s), with a single final extension of 7 min at 72° C.	
efaA	F: AACAGATCCGCATGAATA<br>R: CATTTCATCATCTGATAGTA	50	692	Same as *acm*	55
csuE	F: CATCTTCTATTTCGGTCCC<br>R: CGGTCTGAGCATTGGTAA	56	168	5 min at 94°C, followed by 35 cycles with denaturation at 94°C for 50 s, annealing at 55–57°C for 30 s, extension at 72°C for 30 s, and a final extension at 72°C for 5 min.	56
pgaB	F: AAGAAAATGCCTGTGCCGACCA<br>R: GCGAGACCTGCAAAGGGCTGAT	57	490	94 °C 10 min, 45 sec X30 94°C ,57 °C 45 sec,72 °C 1 min, 72 °C 7 min extension.	57
ompA	F: AGGTCTAGAATGAAATTGAGTCGTATTGC<br>R: CGTGGATCCTTTTTACTGTTCAAGAACTC	61	1112	initial denaturation at 95 C for 5 min, followed by 35 cycles; denaturation at 94 C for 1 min, annealing at 61 C for 1 min and extension at 72 C for 1 min. Ultimately, a final extension phase was programmed at 72 C for 10 min	58
bap	F: ATGCCTGAGATACAAATTAT<br>R: GTCAATCGTAAAGGTAACG	55	1449	1 cycle at 95 °C for 2 min followed by 35 cycles at 95 °C for 25 s, annealing at 55 °C for 5 min,	59

				and extension at 72 °C for 20 s	
epsA	F: TGCGAGTTGTGCAGTTACCTCCG<br>R: GCCAGCTGCTTTATAGCGTCCCA	57	358	initial single cycle at 95°C for 5 min, followed by 35 cycles of melting at 94°C for 1 min, annealing at 57°C for 40 sec, and elongation at 72°C for 45 sec. A final and unique cycle at 72°C for 5 min was included	60
algD	F: TTCCCTCGCAGAGAAAACATC<br>R: CCTGGTTGATCAGGTCGATCT	60	520	30 cycles: 1 min 94°C, 1 min 60°C, 1 min 72°C	37
pslA	F: TCCCTACCTCAGCAGCAAGC<br>R: TGTTGTAGCCGTAGCGTTTCTG	60	680	95°C (5 min) +35X [95°C (1 min) +60°C (1min) +72°C (1 min)] +72°C (5 min)	61
pelA	F: CATACCTTCAGCCATCCGTTCTTC<br>R: CGCATTCGCCGCACTCAG	52	750	94°C (5 min) +35X [94°C (30 sec) +52°C (40 sec) +72°C (50 sec)] +72°C (7 min)	61
mrkA	F: ATGCGAACGTTTACCTGTCTCC<br>R: CCCGGGATGATTTTGTTGG	58	298	95 °C, 30 sec,58 °C, 30 sec,72 °C, 30 sec (33 cycles)	62
mrkD	F: GTCTTTTCGTCCCGGGTATATAAC<br>R: CCACATCGACATTCATATTTTTCC	58	244	*Same as mrkA*	62
luxS	F: TGACTGGCTTCCTGATGC<br>R: GCCGTTGTTAGATAGTTTCACAG	53	427	initial denaturation at 94 °C for 7 min, followed by 33 cycles of denaturation at 95 °C for 30 s, annealing at 53 °C for 60 s, extension at 72 °C for 60 s and a final extension at 72 °C for 5 min.	63

treC	F: GAGCACTTCTTTTGTGATGGC<br>R: CGACAGCGGGCAGTATT	53	71	Same as *LuxS*	63

Supplementary Table S2 Prevalence of biofilm-related genes in this study compared to pan-genomes from PanX (NM = Not mentioned)

Bacteria	Biofilm-related genes percentage								
	clfA	*clfB*	*cna*	*fnbA*	*isdA*	*isdB*	*sdrD*	*sdrE*	*bbp*
S. aureus	100	92.8	100	67.9	78.6	96.4	100	100	39.3
Pan-genome (*S. aureus*)	97	96.4	33.4	96.4	100	99.6	90.8	90	NM
	hyl	*esp*	*asa1*	*gelE*	*acm*	*efaA*			
E. feacium	13.3	6.67	13.33	40	86.7	86.7			
Pan-genome (*E. feacium*)	NM	NM	NM	60.6	86.7	80.3			
	csuE	*bap*	*ompA*	*pgaB*	*espA*				
A. baumannii	97.1	37.1	100	82.9	90				
Pan-genome (*A. baumannii*)	91.2	NM	100	98.5	NM				
	algD	*pslA*	*pela*						
P. aeruginosa	88.6	100	100						
Pan-genome (*P. aeruginosa*)	99.6	87.4	95.6						
	fimH	*mrkA*	*mrkD*	*luxS*	*treC*				
K. pneumonia	40	34.29	20	100	71.43				
Pan-genome (*K. pneumonia)*	0	93.6	97.4	100	99.6				
